# The psychometric properties of the Comprehensive Headache-related Quality of life Questionnaire (CHQQ) translated to Serbian

**DOI:** 10.1186/s40064-016-3109-1

**Published:** 2016-08-24

**Authors:** Slobodan M. Jankovic, Marija Andjelkovic, Radica Zivkovic Zaric, Marko Vasic, Éva Csépány, Tamás Gyüre, Csaba Ertsey

**Affiliations:** 1 Faculty of Medical Sciences, University of Kragujevac, Kragujevac, Serbia; 2 János Szentágothai Doctoral School of Neurosciences, Semmelweis University, Budapest, Hungary; 3 Department of Neurology, Faculty of General Medicine, Semmelweis University, Balassa Str. 6, Budapest, 1083 Hungary

**Keywords:** Headache, Migraine, Quality of life, Questionnaire

## Abstract

**Background:**

The Comprehensive Headache-related Quality of life Questionnaire (CHQQ), is a recently developed and validated instrument, intended for measuring quality of life of patients with all headache types. Currently no validated headache-specific quality of life questionnaires are available in Serbian. The aim of this study was to translate the CHQQ from Hungarian to Serbian, to make necessary cultural adaptations and to test its psychometric properties in a sample of outpatients with headache.

**Methods:**

The CHQQ was translated and adapted according to internationally accepted guidelines, and then tested on a sample of 216 Serbian headache patients (171 females and 45 males, mean age 42.3 years/SD 13.35; range 18–75). The majority of patients suffered from episodic tension-type headache (TTH); 27 (12.5 %) had episodic migraine. We calculated the internal consistency (Cronbach’s alpha), criterion validity (correlations of individual items, dimensions and whole questionnaire with the clinical characteristics of headache), convergent validity (correlations of the abovementioned scores with results of other instruments measuring headache severity and impact), and discriminative validity (comparison of the scores in the two diagnostic groups) of the CHQQ. We used factor analysis to explore the underlying construct.

**Results:**

The Serbian translation of CHQQ showed excellent internal consistency, both for the whole instrument (Cronbach’s alpha 0.937) and its dimensions. The validity of the instrument in all aspects (criterion, convergent and discriminative validity) was also excellent when the whole sample and the subgroup of patients with TTH were analyzed, while the results for patients with migraine were less favorable. Factor analysis suggested the existence of a single dimension in this sample.

**Conclusions:**

The Serbian translation of CHQQ is as reliable and valid specific instrument for measuring headache-related quality of life in patients with TTH and probably in patients with migraine.

## Background

Headache is one of the most prevalent disorders in almost all age groups all over the world and is a prominent cause of disability. In the latest edition of the Global Burden of Disease Study, migraine alone was the 6th highest cause of disability worldwide, and medication overuse headache was the 18th, making headache disorders the third (!) most important cause of worldwide disability (Steiner et al. 2015). This, in part, is explained by the high prevalence of headache disorders: among the adults the 1-year prevalence of migraine is 10–18 %, and that of tension-type headache is 31–90 % (Zebenholzer et al. 2015). Patients suffering from headache lose a lot of workdays and cannot engage in many social, family or leisure activities, all of which have substantial negative impact on quality of life. Headaches, especially chronic headaches, also have a significant psychiatric comorbidity with increased risk of anxiety, depression, panic disorder, and suicide attempts, as reviewed in Pompili et al. (2010). The comorbidity with these psychiatric conditions may lead to less favourable outcomes and has a negative influence on the perceived quality of life and disability of the sufferers (Lanteri-Minet et al. 2005; Risal et al. 2016). The treatment of headache is nowadays targeted not only towards absence of pain, but to overall improvement in quality of life (Freitag and Schloemer 2014).

Measuring quality of life (QOL) in patients with headache is important for research purposes and is frequently used as a secondary endpoint in interventional studies. Assessing QOL may also be important because it reports the subjective experience of the patient, giving a unique insight into the way the headache would affect the patient’s daily life. In this respect, QOL instruments may complement the information of the patients’ conditions given by headache diaries and headache severity scales.

There are several specific instruments for measuring quality of life in patients with headache, but the majority of them were developed for use in patients with migraine, leaving patients with other headache types without a suitable tool. This limitation was recently overcome by development and validation of the Comprehensive Headache-related Quality of life Questionnaire (CHQQ) which is intended for use in all headache types (Manhalter et al. 2012). The CHQQ contains 23 items probing various aspects of daily life that had been found important by a panel of headache experts and patients (Manhalter et al. 2012). Based on the 23 items, three dimensions (physical, mental and social) and a total score are calculated and transformed to a 0–100 scale (100 representing the best possible QOL) as is the usual practice in reporting QOL. The original Hungarian CHQQ scale showed adequate reliability, criterion and convergent validity in migraineurs and tension type headache patients (Manhalter et al. 2012), with migraineurs having significantly lower scores (worse QOL) in 16 of the 23 items, all dimensions and total score, as expected. The preliminary results of CHQQ’s validation in 60 patients suffering from cluster headache were also encouraging, with excellent reliability and adequate criterion and convergent validity (Ertsey et al. 2015a). The CHQQ was recently found to be responsive to the effect of treatment in a pilot study of patients suffering from medication overuse headache (Gyüre et al. 2014). Taken together, the CHQQ seems to be suitable for every-day use in clinical practice.

The current study reports the first validation effort of CHQQ in a foreign language. At present, there are no cross-culturally validated versions of CHQQ in other languages: translations in English, Farsi and Urdu are being tested in the USA, India, Iran and Pakistan, respectively (Ertsey et al. 2015b).

Currently, there are no available Serbian translations with trans-cultural adaptations of headache-specific quality of life measurement instruments, so researchers in Serbia use only basic clinical tools when investigating patients with headache (Stojimirovic et al. 2015).

The aim of our study was to translate the Comprehensive Headache-related Quality of life Questionnaire (CHQQ) from Hungarian to Serbian, to make necessary cultural adaptations and to test its psychometric properties in a sample of outpatients with headache.

## Patients and methods

### Patients

The study included outpatients of a primary care Health Center in Kragujevac, Serbia, who consecutively visited their general practitioners from February to June 2015. We only included patients who had previously been diagnosed by a neurologist either with episodic migraine (with or without aura; ICHD-II codes 1.1 and 1.2) or (with episodic tension-type headache; ICHD-II code 2.1) (Headache Classification Subcommittee of the International Headache Society 2004). The patients with the following diagnoses were excluded from the study: rare adult migraine subtypes, probable migraine, probable tension-type headache, analgesic abuse, moderate to severe liver or kidney failure, untreated hypertension, refractive disorders and chronic pain syndromes. Patients with mood or anxiety disorders were not excluded from the study, but psychiatric comorbidities were not formally tested and therefore were not applied as grouping variables.

All patients gave informed consent to participating in the study. The study was approved by the Ethics Committee of the Health Center in Kragujevac, Serbia, where the study took place.

A total of 216 patients completed all the questionnaires and were included in the analysis. There were 171 females (79.2 %) and 45 males (20.8 %), all of Caucasian origin. The mean age was 42.3 years, (SD 13.35; range 18–75). One hundred and sixty-nine patients (78.2 %) were employed, whereas 21 (9.7 %) were unemployed, 15 (6.9 %) were retired and 11 (5.1 %) were students.

The majority of our patients suffered from episodic tension-type headache (189 patients, i.e. 87.5 %), and 27 (12.5 %) were migraineurs. The patients with migraine had a somewhat higher attack frequency (mean 11.9 attacks in 3 months, SD 9.2, vs. 9.8 in 3 months, SD 14.2, p > 0.05), significantly higher pain intensity (mean 7.9 points on a scale from 1 to 10, SD 1.7, vs. 5.5 points, SD 2.2, p < 0.01) and significantly longer duration of attack (mean 619.6 min, SD 1002.6 min, vs. 281.7 min, SD 524.3 min, p < 0.01) than patients with tension-type headache. The ratio of males to females was not significantly different among the patients with migraine and among those with tension-type headache (2:25 vs. 43:146, Fisher’s exact test p = 0.078). Only 3 patients reported medically treated depression as concomitant condition, and all of them suffered from tension-type headache.

### Methods

#### Translation and cultural adaptation of the original CHQQ questionnaire

The translation and cultural adaptation of the CHQQ was made according to the guidelines of the International Society for Pharmacoeconomics and Outcomes Research (ISPOR) (Wild et al. 2005). The original scale was first translated from Hungarian to Serbian by two independent Serbian persons, a professional translator and a bilingual university lecturer. They translated the scale independently of each other, and then the translations were harmonized to one Serbian version at the meeting of the study investigators and the translators. The harmonized Serbian version was then translated back to Hungarian by a native Hungarian translator, who was not aware of the original Hungarian version of the CHQQ. The back-translation to Hungarian was then compared with the original Hungarian version by the study investigators, and at new meeting of investigators the final Serbian version of the CHQQ was agreed on. The final translation of CHQQ to Serbian was then tested on 10 headache patients at the Health Center in Kragujevac, Serbia for clarity and comprehension. After the pilot a few minor changes of wording were made, and then final Serbian version of the CHQQ was copied and prepared for psychometric testing.

#### Data recording

The patients completed the Serbian version of CHQQ, the Serbian version of the Headache Under-Response to Treatment (HURT) Questionnaire (Buse et al. 2012) and the Serbian version of The Migraine Disability Assessment Test (MIDAS) (Stewart et al. 2001) in the Health Center, after their encounter with the general practitioners, who administered the questionnaires. After completing the questionnaires, the patients returned them to the general practitioners. The general practitioners collected the clinical characteristics of the patients during the encounter, using a purpose-built checklist. Headache severity was assessed by the patient, using a visual analogue scale (VAS; 0–100 mm). Headache diagnoses had previously been made by neurologists, and for the purpose of this study were copied from the patients’ files. The patients were not formally tested for depression or anxiety.

### Statistical analysis

Kolmogorov–Smirnov tests with Lilliefors correction were used to test whether the questionnaires’ data were normally distributed. As most of the data were not normally distributed, we used nonparametric tests where applicable.

The reliability of the Serbian version of CHQQ was tested by measuring the internal consistency through calculation of the Cronbach’s alpha. The Cronbach’s alpha was calculated for both the whole instrument and its dimensions. The criterion validity of the Serbian version of CHQQ was tested by the correlation of the patient’s headache characteristics with the individual items, dimensions and total score of the questionnaire. The convergent validity was assessed through investigation of correlation of the individual items, the three dimensions and the total score with the total score of the HURT and MIDAS instruments. We calculating Spearman’s rank correlations for all tests of criterion and convergent validity. The discriminative validity was tested by comparing the results of the Serbian version of CHQQ in the two diagnostic groups, the patients with migraine and tension-type headache, by means of the Mann–Whitney test.

As the data were not normally distributed, the factor structure of the Serbian translation of CHQQ was examined by the principal axis factoring method, as recommended by Fabrigar et al. (1999). The factors were extracted with the condition that Eigenvalues had to be greater than 1. Extracted factors were than compared with their respective CHQQ items, and named accordingly.

All calculations in this study were performed by SPSS software, version 18. The level of significance was set to p < 0.05.

## Results

### Completing the questionnaires

The average duration of completing all the questionnaires of the study was less than 25 min, and the patients did not complain about any difficulty. All patients answered to each of the questions in the three questionnaires. Therefore, the answers of all 216 patients were entered to the statistical calculations of reliability and validity.

### Reliability

The reliability of the questionnaire when tested on the whole study sample was very good, with the Cronbach’s alpha being 0.937; when tested on patients with tension headache only, the Cronbach’s alpha was 0.930, and the same parameter was 0.954 in the group of migraineurs. The dimensions of the questionnaire (physical, mental and social) also showed satisfactory reliability in both the whole study sample and in the diagnostic subgroups (Table 1).

**Table 1:** The internal consistency of the questionnaire and its dimensions (Cronbach’s alpha values)

	Whole study sample	Patients with TTH	Patients with migraine
Total score	0.937	0.930	0.954
Physical dimension	0.915	0.914	0.870
Mental dimension	0.835	0.814	0.896
Social dimension	0.722	0.692	0.888

### Validity

#### Criterion validity

When taking the whole sample, all 23 items, total questionnaire score, and scores of physical, mental and social dimensions were significantly and negatively correlated with headache frequency and headache severity. It was similar with mean attack length, with the exception of the item 21 (embarrassment due to headaches) where the correlation was not significant. On the other hand, disease length correlated with the dimensions and the total score, but only with 14 of the 23 the items (items nos. 1–6, 8, 9, 11, 16–19, and 22). The results were similar in the subgroup of patients with tension-type headache. However, in the subgroup of patients with migraine, the dimensions and the total score correlated negatively and significantly only with headache severity (Table 2).

**Table 2:** The criterion validity of the questionnaire: the correlations of the items, dimensions and total score of the instrument with the clinical characteristics

Item or score	Group	Frequency of headache attacks (in the last 3 months)	Headache severity (VAS)	Mean attack length (min)	Disease length (years)
1. Work performance	ALL	*−0.467*	*−0.527*	*−0.242*	*−0.207*
2. Household chores	ALL	*−0.487*	*−0.547*	*−0.237*	*−0.168*
3. Social life	ALL	*−0.502*	*−0.528*	*−0.279*	*−0.217*
4. Leisure activities	ALL	*−0.491*	*−0.550*	*−0.323*	*−0.247*
5. Vacations/awaydays	ALL	*−0.496*	*−0.522*	*−0.273*	*−0.167*
6. Physical health	ALL	*−0.537*	*−0.520*	*−0.285*	*−0.261*
7. Appearance	ALL	*−0.400*	*−0.477*	*−0.208*	−0.128
8. Relationship with other family members	ALL	*−0.391*	*−0.440*	*−0.213*	*−0.164*
9. Sexual life	ALL	*−0.379*	*−0.414*	*−0.258*	*−0.223*
10. Sleep	ALL	*−0.458*	*−0.461*	*−0.234*	−0.048
11. Energy	ALL	*−0.453*	*−0.552*	*−0.333*	*−0.148*
12. Mood	ALL	*−0.424*	*−0.526*	*−0.278*	−0.126
13. Memory	ALL	*−0.354*	*−0.304*	*−0.157*	−0.076
14. Concentration	ALL	*−0.463*	*−0.427*	*−0.235*	−0.084
15. Thinking	ALL	*−0.470*	*−0.407*	*−0.210*	−0.065
16. General health perceptions	ALL	*−0.382*	*−0.458*	*−0.181*	*−0.137*
17. Irritability	ALL	*−0.441*	*−0.486*	*−0.246*	*−0.279*
18. Frustration	ALL	*−0.438*	*−0.481*	*−0.327*	*−0.197*
19. Abortive medication use	ALL	*−0.397*	*−0.520*	*−0.311*	*−0.258*
20. Financial situation	ALL	*−0.281*	*−0.305*	*−0.144*	−0.084
21. Embarrassment due to headaches	ALL	*−0.155*	*−0.143*	*−*0.002	0.036
22. Worries about headache	ALL	*−0.530*	*−0.587*	*−0.290*	*−0.142*
23. Life enjoyment	ALL	*−0.450*	*−0.565*	*−0.280*	−0.118
Physical score	ALL	*−0.558*	*−0.615*	*−0.313*	*−0.221*
	TTH	*−0.567*	*−0.575*	*−0.299*	*−0.160*
	Migraine	*−*0.138	*−0.614*	*−*0.040	−0.143
Mental score	ALL	*−0.577*	*−0.606*	*−0.337*	*−0.176*
	TTH	*−0.569*	*−0.578*	*−0.319*	*−*0.112
	Migraine	*−*0.253	*−0.471*	*−*0.111	*−*0.037
Social score	ALL	*−0.542*	*−0.572*	*−0.314*	*−0.193*
	TTH	*−0.541*	*−0.555*	*−0.307*	*−0.158*
	Migraine	−0.197	*−0.511*	−0.079	−0.059
Total score	ALL	*−0.584*	*−0.628*	*−0.337*	*−0.206*
	TTH	*−0.586*	*−0.599*	*−0.324*	*−0.146*
	Migraine	−0.187	*−0.577*	−0.015	−0.044

* ALL, whole sample, TTH, subgroup of patients with tension-type headache; Migraine, subgroup of patients with migraine; values of Spearman’s correlation coefficients given in italic are significant (p < 0.05)

#### Convergent validity

The CHQQ instrument showed excellent convergent validity for the whole sample and for the subgroup of patients with tension-type headache (Table 3). All items, the total score of CHQQ and the scores of the dimensions correlated negatively and significantly (with high correlation coefficients) with both MIDAS and HURT scores. The results were less favorable for the subgroup of patients with migraine, where only the mental dimension correlated both negatively and significantly with the HURT score, while the total score of CHQQ and the scores of other dimensions correlated negatively, but not significantly with MIDAS and HURT scores (Table 3).

**Table 3:** The convergent validity of the questionnaire the correlations of the items, dimensions and total score of the instrument with the total scores of the Headache Under-Response to Treatment (HURT) Questionnaire and The Migraine Disability Assessment Test (MIDAS)

Item or score	Group	MIDAS	HURT
1. Work performance	ALL	*−0.609*	*−0.542*
2. Household chores	ALL	*−0.623*	*−0.552*
3. Social life	ALL	*−0.608*	−0.528
4. Leisure activities	ALL	*−0.557*	*−0.474*
5. Vacations/awaydays	ALL	*−0.562*	*−0.581*
6. Physical health	ALL	*−0.606*	*−0.552*
7. Appearance	ALL	*−0.507*	*−0.439*
8. Relationship with other family members	ALL	*−0.532*	*−0.433*
9. Sexual life	ALL	*−0.551*	*−0.383*
10. Sleep	ALL	*−0.451*	*−0.466*
11. Energy	ALL	*−0.547*	*−0.492*
12. Mood	ALL	*−0.556*	*−0.493*
13. Memory	ALL	*−0.415*	*−0.399*
14. Concentration	ALL	*−0.566*	*−0.481*
15. Thinking	ALL	*−0.509*	*−0.493*
16. General health perceptions	ALL	*−0.509*	*−0.480*
17. Irritability	ALL	*−0.510*	*−0.463*
18. Frustration	ALL	*−0.576*	*−0.537*
19. Abortive medication use	ALL	*−0.501*	*−0.406*
20. Financial situation	ALL	*−0.364*	*−0.357*
21. Embarrassment due to headaches	ALL	*−0.176*	*−0.235*
22. Worries about headache	ALL	*−0.569*	*−0.597*
23. Life enjoyment	ALL	*−0.584*	*−0.559*
Physical score	ALL	*−0.682*	*−0.609*
	TTH	*−0.691*	*−0.626*
	Migraine	−0.221	−0.193
Mental score	ALL	*−0.678*	*−0.632*
	TTH	*−0.677*	*−0.623*
	Migraine	−0.207	*−0.450*
Social score	ALL	*−0.629*	*−0.596*
	TTH	*−0.629*	*−0.598*
	Migraine	−0.225	−0.366
Total score	ALL	*−0.690*	*−0.632*
	TTH	*−0.692*	*−0.634*
	Migraine	−0.211	−0.366

* ALL, whole sample; TTH, subgroup of patients with tension-type headache; Migraine, subgroup of patients with migraine; values of Spearman’s correlation coefficients given in italic are significant (p < 0.05)

#### Discriminative validity

Comparing the CHQQ results among the two subgroups (migraine and TTH) we found showed higher scores in the group of TTH patients. The difference was significant for 20 of the 23 items (7/8 items of the physical dimension, 9/10 items of mental dimension and 4/5 items of the social dimension), and also significant for the whole questionnaire and for each of the three dimensions (Table 4). Comparing the patients’ scores with those of the patients of the original validation study, the Serbian TTH patients had numerically higher CHQQ values on 15/23 items, all dimensions and total score than the Hungarians, while Serbian migraineurs had numerically higher CHQQ values on 14/23 items, 2/3 dimensions and total score than the Hungarians; most divergent items were part of the Mental dimension in both diagnostic groups.

**Table 4:** The discriminative validity of the questionnaire: scores for individual items, dimensions and whole CHQQ questionnaire in the tension-type headache group versus the migraine group

Item or score	TTH (mean ± SD)	Migraine (mean ± SD)	p*
1. Work performance	61.77 ± 26.98	43.52 ± 27.38	*0.002*
2. Household chores	61.77 ± 27.48	38.89 ± 27.15	*0.000*
3. Social life	61.64 ± 28.54	42.59 ± 24.82	*0.001*
4. Leisure activities	56.48 ± 60.76	37.04 ± 28.05	*0.000*
5. Vacations/awaydays	70.77 ± 32.03	46.30 ± 35.15	*0.001*
6. Physical health	60.32 ± 30.39	37.04 ± 28.90	*0.000*
7. Appearance	59.13 ± 32.30	32.41 ± 29.27	*0.000*
8. Relationship with other family members	62.96 ± 29.92	46.30 ± 29.17	*0.006*
9. Sexual life	68.39 ± 32.35	44.44 ± 30.48	*0.000*
10. Sleep	60.58 ± 31.16	45.37 ± 34.69	*0.019*
11. Energy	49.07 ± 46.01	27.78 ± 24.35	*0.000*
12. Mood	47.49 ± 31.00	24.07 ± 22.45	*0.000*
13. Memory	68.25 ± 26.12	54.63 ± 34.69	*0.046*
14. Concentration	54.37 ± 61.97	36.11 ± 29.69	*0.001*
15. Thinking	61.90 ± 26.99	45.37 ± 31.04	*0.009*
16. General health perceptions	66.93 ± 29.92	60.19 ± 29.63	0.238
17. Irritability	64.02 ± 29.88	38.89 ± 32.03	*0.000*
18. Frustration	50.40 ± 28.77	21.30 ± 23.72	*0.000*
19. Abortive medication use	41.27 ± 30.89	14.81 ± 25.25	*0.000*
20. Financial situation	70.11 ± 44.13	68.52 ± 37.08	0.909
21. Embarrassment due to headaches	87.96 ± 38.82	84.26 ± 29.54	0.489
22. Worries about headache	60.71 ± 32.22	38.89 ± 28.87	*0.001*
23. Life enjoyment	64.15 ± 29.31	42.59 ± 24.82	*0.000*
Physical dimension	60.02 ± 23.87	39.58 ± 21.12	*0.000*
Mental dimension	60.83 ± 22.42	41.39 ± 20.38	*0.000*
Social dimension	64.39 ± 27.39	48.15 ± 25.91	*0.000*
Total score	*61.32* *±* *22.37*	*42.23* *±* *20.62*	*0.001*

* TTH, subgroup of patients with tension-type headache; Mann–Whitney tests; significant differences (p < 0.05) are marked in italic

### Factor structure of the questionnaire

In order to check whether dimensions of the questionnaire defined in the Hungarian version correspond to the dimensions (factors) in Serbian version, we performed exploratory factor analysis with principal axis factoring of the whole sample and on the diagnostic subgroups. When examining the whole sample, a single factor emerged, explaining 56.1 % of the total variance. Nineteen of the 23 items had high (>0.7) loadings on this factor; exceptions were items 13, 19, 20 and 21. As this factor encompasses items with very different facets of quality of life, it could be called ‘, general effects of headache’. In the TTH subgroup, the factor structure was very similar, with a single factor explaining 54.9 % of the total variance: seventeen of the 23 items had high (>0.7) loadings on this factor, with the exception of items 9, 13, 17, 19, 20 and 21. In the group of migraineurs, two factors emerged, explaining 52.2 and 8.5 % of the total variance, respectively. Thirteen items had high (>0.7) loadings on factor 1, including items 1–6, 8, 9, 11, 12, 15, 22 and 23. Two items (20 and 21) had high loadings on factor 2.

## Discussion

This study is the first to report the psychometric properties of the headache-specific quality of life CHQQ instrument in a foreign language. The Serbian translation of CHQQ showed excellent internal consistency, both for the whole instrument and its individual dimensions. The validity of the instrument was also excellent in all aspects (criterion, convergent and discriminant validity) when whole sample and subgroup of patients with TTH were analyzed; only two items, i.e. item 20 (financial situation) and item 21 (embarrassment due to headaches) did not perform well. The lack of significant correlations in the case of these two items can probably be explained with cultural and social differences. The attitude towards patients in Serbia is generally sympathetic and respectful (Jocić and Krajnović 2014), so they are rarely embarrassed by their disease, and this may be even more true in case of episodic headaches, which do not cause permanent or readily perceptible hindrances. Besides, the majority of patients in Serbia are paid minimal wages, therefore they do not perceive transient absences from work as a major financial loss (Stosić and Karanović 2014). This was probably the reason why these two items did not correlate with severity and headache frequency well, and why their impact on quality of life was small.

The psychometric properties of the CHQQ were less satisfactory in the subgroup of patients with migraine. The correlation coefficients of the scale scores with headache frequency, severity and attack duration were relatively high and negative, as expected, but significance was not reached. The correlations of migraineurs’ CHQQ scores with their respective MIDAS and HURT scores were also in the expected direction, but they were not significant. This is not necessarily the consequence of an inherent weakness of the instrument: in fact, CHQQ’s original version had showed significant correlations with the clinical data and SF-36 scores of migraineurs (Manhalter et al. 2012). The main reason of not finding significant correlations in the current sample was probably the small number of migraineurs (27 patients). Further investigations, involving a significantly bigger number of Serbian migraine patients are necessary to assess the validity of CHQQ in migraine.

When compared to the original Hungarian version (Manhalter et al. 2012), the Serbian translation of CHQQ is non-inferior, and in some aspects even shows better results. The internal consistency of the Serbian translation was slightly higher, and correlations with the headache characteristics were stronger than in the original Hungarian sample. The convergent validity was especially impressive in the Serbian sample, since most of the negative correlations with HURT and MIDAS were higher than 0.5. The Serbian translation of CHQQ would perform even better if the items 20 and 21 (which are probably not relevant for the patients due to cultural and social differences) were excluded: after elimination of these two items the Cronbach’s alpha for the whole Serbian sample would grow from 0.937 to 0.944. However, both with and without the items 20 and 21, the Serbian translation of the CHQQ can be considered as reliable and valid instrument for measurement of headache-related quality of life in patients with TTH and migraine. This finding is especially interesting if one considers the linguistic differences: while the original version of the CHQQ was developed in Hungarian (a Finno-Ugric language), the present study was done in Serbian (a Slavic language), so this study underlines that the results of the original validation study may be replicated in other languages (belonging to different language families), and indicates that the CHQQ may be used in different linguistic or cultural settings, too.

The original validation study of the CHQQ was performed on a group of consecutive patients visiting a headache specialty service. As expected, most of the patients had migraine, but the study also included 34 patients with TTH (Manhalter et al. 2012). Although that study found that the CHQQ was reliable and valid in TTH as well, the criterion and convergent validity in the TTH group were less robust than those in the migraine group. In the present study, most of the patients had tension-type headache, the effect of which on quality of life had less often been studied. The results of the present study confirm that CHQQ can be used for studying quality of life in tension type headache as well.

Similarly to other QOL instruments, the items of the original CHQQ were grouped in three dimensions (physical, mental and social), which was based on the content of the items. Item-dimension correlations supported the hypothesized dimensions, but a factor analysis was not performed on the Hungarian sample of patients (Manhalter et al. 2012; Manhalter et al. 2010). The factor analysis of the current Serbian translation confirmed the existence of a single factor (dimension). Nineteen of the 23 items had high (>0.7) loadings on this factor; exceptions were items 13, 19, 20 and 21. Interestingly, items conceptually belonging to physical, mental and social aspects of QOL all loaded on this factor, which therefore could be called ‘general effects of headache’. The factor analysis performed on the TTH patients showed a remarkably similar picture, with two more items (9 and 17) not having sufficient loadings on this factor. On the other hand, the factor analysis of the migraine patients showed an additional factor with significant loadings of items 20 and 21. Interestingly, these are the items that may have a smaller effect on Serbian patients’ QOL due to sociocultural reasons, as discussed above. However, due to the small number of migraine patients in our sample, drawing conclusions about the factor structure of CHQQ in migraine would not be legitimate.

Apart from interpretation restrictions due to the sample size (see below), the single-factor solution of the factor analysis of the whole sample and also of the TTH patients is not entirely unexpected. A quite similar result was found in a study about the psychometric properties of the Stagnation Scale in medication overuse headache patients (Innamorati et al. 2015), where structural equation modeling of the data indicated the presence of a general factor common to all the items of the Stagnation Scale and three specific latent factors underlying groups of its items. Performing structural equation modeling of CHQQ on a larger Serbian dataset could help us understand whether, apart from the common factor, other latent factors are at play.

The main limitation of the present study is the sample size, which calls for the cautious interpretation of our findings in two instances. First, the ratio of the total number of subjects and the items in the CHQQ is 9.4, adding an element of uncertainty about the legitimacy of performing factor analysis. In a paper about the best practices in exploratory factor analysis, the authors cite a frequently used rule of the thumb requiring a 10 or higher subject:item ratio. Interestigly, this requirement was not met by more than 60 % of 303 research articles surveyed by the authors (Costello and Osborne 2005). As the present study also fails to reach an adequate subject:item ratio (and this ratio is even smaller in the two diagnostic groups), our results about the factor structure of the CHQQ may not reflect the true structure and should be verified on samples of an adequate size.

We therefore suggest that in further studies of the CHQQ, both the original (3-dimension) and the above single-dimension structures should be examined. The agreement about the final number of dimensions of the CHQQ should await further studies in other populations and headache types, with an adequate number of patients in all diagnostic subgroups.

The second limiting aspect of the sample size is the small number of migraineurs, which precluded gathering a complete picture about performance of the Serbian translation in patients with migraine. This limitation was caused by the site where the study was performed: as opposed to most validation studies, which had been carried out in tertiary headache centers, the present study was performed in a primary healthcare facility, involving patients who had been diagnosed with either migraine or tension type headache by a neurologist, but not necessarily presenting because of their headache problem. While further testing of the CHQQ in Serbian migraineurs is necessary to conclude its validation for migraine, and studies in tertiary centres may profit from a higher probability of correct diagnosis, we think the primary care setting is actually an advantage in two regards. First, the proportions of TTH and migraine in the sample reflect the significantly higher prevalence of TTH than that of migraine in general population (Zebenholzer et al. 2015; Vlajinac et al. 2004). Second, the results are probably less affected by the selection bias that one is likely to have when studying patients in a tertiary headache center (where patients with more severe conditions are probably overrepresented), and the CHQQ results are probably closer to those of an ‘average’ patient suffering from TTH or migraine, respectively. The difference between the settings of the present and the Hungarian validation study may also explain why Serbian patients (both the migraine and TTH groups) had numerically higher CHQQ scores than their Hungarian counterparts.

A further limitation of our study is the fact that we did not formally check depression and anxiety in the sample. Although three patients reported medically treated depression, previous research suggests that the prevalence of underlying psychopathology is much higher in headache sufferers (Pompili et al. 2010), and this is probably true also for our sample. In similar validation studies (Seo and Park 2015a; b) the prevalence of major depressive disorder and generalized anxiety disorder were found to be 29 and 22 %, respectively. Health and QOL perceptions of headache patients also suffering from depression or anxiety disorder seem to be less favorable than those of patients not harboring any psychopathology (Lanteri-Minet et al. 2005; Risal et al. 2016). In fact, depression emerged as the strongest predictor in a study analyzing the effect of mood disorders, disability, clinical and psychosocial factors on QOL (Kim and Park 2014). This should be taken into account when interpreting our data. On the other hand, the psychological testing of our patients would either have meant face-to-face interviews, or further increasing responder burden by adding validated questionnaires. None of these seemed feasible to us.

In conclusion, with the above discussed limitations, the current study indicates that the Serbian translation of CHQQ is a reliable and valid specific instrument for measuring headache-related quality of life in patients with TTH and may have adequate psychometric properties in patients with migraine. Further studies are necessary to confirm the usefulness of this instrument in Serbian patients suffering from migraine, and to explore its suitability for other types of headache.
